# Physiotherapists’ adherence to Clinical Practice Guidelines in fibromyalgia: a cross-sectional online survey

**DOI:** 10.1007/s00296-024-05630-4

**Published:** 2024-06-05

**Authors:** José Édgar Ferrández-Gómez, Mariano Gacto-Sánchez, Rauf Nouni-García, Jaime Gascón-Jaén, Carlos Lozano-Quijada, Aitor Baño-Alcaraz

**Affiliations:** 1 Physiotherapy Area, Pathology and Surgery Department, School of Medicine, University of Miguel Hernández de Elche, Ctra, Nacional N-332 s/n, 03550 San Juan de Alicante, Spain; 2 Faculty of Physiotherapy, Occupational Therapy and Podiatry, UCAM Catholic University of Murcia, Murcia, Spain; 3 Department of Physical Therapy, Campus of Health Sciences, University of Murcia, Av. Buenavista, 32 El Palmar, 30120 Murcia, Spain; 4 Diagnostic Center, Institute of Health and Biomedical Research of Alicante, General University Hospital of Alicante, Fifth Floor, Pintor Baeza Street, 12, 03110 Alicante, Spain; 5 Network for Research on Chronicity, Primary Care and Health Promotion (RICAPPS), 03550 San Juan de Alicante, Spain; 6 Department of Pathology and Surgery, Faculty of Medicine, Center for Translational Research in Physiotherapy, Miguel Hernandez University, Ctra. Alicante-Valencia Km. 8,7—N 332, 03550 Alicante, Spain

**Keywords:** Fibromyalgia, Guideline adherence, Physical therapy modalities, Health education, Chronic pain, Surveys and questionnaires

## Abstract

To evaluate the level of knowledge and adherence to Clinical Practice Guidelines on fibromyalgia of physiotherapists in Spain. A cross-sectional study using an ad-hoc online survey was implemented to assess aspects on the assessment, treatment, and decision of the length of the therapeutic approach on fibromyalgia. Based on the results, professionals were classified as adherent, partially adherent, or non-adherent. The level of agreement with several statements on the condition was also evaluated across the professionals surveyed to evaluate the potential consensus. A total of 240 physiotherapists met inclusion criteria, amongst which 68 (28.33%) were adherent. The academic level of studies (Chi-square = 48.601, p-value = 0.001) and having had previous training in fibromyalgia (Chi-square = 151.011, p-value = 0.001) displayed statistically significant differences across adherence-based groups. Consensus was reached for 15 out of 24 statements. Our findings highlight the presence of an acceptable level of knowledge and adherence to clinical practice guidelines in the field of fibromyalgia among physiotherapists in Spain.

Practice implications

Our results also reveal the existence of an evidence-to-practice gap in the field, with potential room for improvement: further efforts on promoting and reinforcing the importance of evidence-based therapies are needed, from university teaching plans to clinical updates for daily practice.

**Supplementary Information:**

The online version contains supplementary material available at 10.1007/s00296-024-05630-4.

## Background

Fibromyalgia (FM) is the third most common musculoskeletal condition worldwide, after lumbar pain and osteoarthritis [1], with a global prevalence of 2.7%. It is more common in women than in men, with a 3:1 ratio [2]. In Europe, the prevalence is 2.3% whilst, in Spain, these figures correspond to 2.4%, and peaked in a 60-to-69 years range [3]. FM is considered as a nociplastic pain condition [4]. It is characterized by chronic widespread pain, fatigue, and sleep disorders, accompanied by other symptoms (autonomic disturbances, cognitive dysfunction, hypersensitivity to external stimuli, somatic symptoms, and psychiatric disorders) [2]. Despite the fact that the etiopathogenesis of FM remains unclear, an amplified processing of and/or decreased inhibition of nociceptive stimuli at multiple levels in the nervous system has been stated [2, 5]. This dysregulation of the nociceptive system may be a product of a combination of interactions between neurotransmitters, cytokines, hormones, the autonomic nervous system, behavioral constructs, and external stressors [6]. All these complex symptoms may have an impact on daily life activities for the subjects affected by FM, decreasing their quality of life and causing changes in living habits and daily routines [7]. According to the American College of Rheumatology (ACR) [8], the diagnosis of FM is based on four core points: (1) Patient’s identification of generalized pain in, at least, 4 out of 5 regions; (2) Symptoms have been present at a similar level for, at least, 3 months; (3) Score in the Widespread Pain Index (WPI) ≥ 7 points and Symptom Severity Scale (SSS) ≥ 5 points, or WPI of 4–6 and SSS ≥ 9; In addition, (4) a diagnosis of FM does not exclude other clinically important conditions. One of the main reasons behind the therapeutic failure in the management of FM is the unsatisfactory or deficient communication between patient and the healthcare provider, fact that lessens or hinders patient’s adherence [9].

In the past decades, several clinical practice guidelines (CPGs) have been published with the aim of providing evidence-based recommendations for the management of FM, from the initial approach by the American Pain Society (APS) in 2004 [10] to the most recent publications by the Italian Rheumatology Society (SIR) [11] and the European Alliance of Associations for Rheumatology (EULAR) [12]. A common approach shared by the different CPGs published is that the treatment should focus on improving health-related quality of life, whilst the first line treatment should be non-pharmacological management considering dimensions as availability, cost, safety issues and patient preference [11, 12]. Education, understood as providing information about the disease and certainty about the prognosis, is considered the core of the treatment [12]. Patients usually express that better self-understanding their condition would lead to greater well-being [13], and that, conversely, the experience of invalidation and the inability to receive answers may have a negative impact on health-related outcomes as anxiety and/or depression [14]. Moreover, psychological therapies as behavioral-cognitive therapy or mindfulness-based stress reduction programs could be used as a part of multicomponent treatment, whether in cases of poor levels of initial improvement, or in patients with mood disorders (e.g. pain-related depression or catastrophizing) [12]. These strategies are not only effective in improving depression, anxiety, or sleep disorders but, moreover, they channel the adoption of an active role in the management of the pathology from the patient, which is of paramount importance [15–17]. Thus, a common characteristic shared by CPGs is that the therapeutical approach of FM should be patient-centered, even though the latest publications evince that that FM is not always addressed in this manner [18].

Concerning the pharmacological approach, pregabalin, and duloxetine are considered the most effective drugs for the management of FM, due to both their effects in improving the symptoms derived (e.g., pain or depression) and the low rate of adverse effects caused [19]. Also, amitriptyline and cyclobenzaprine are effective in improving sleep quality, fatigue, and the general quality of life [20, 21], whereas Nonsteroidal Anti-Inflammatory Drugs (NSAIDs) and paracetamol are no longer recommended in the therapeutic approach of primary pain disorders, including FM [11, 12].

Focusing on the specific physiotherapy-based management included in CPGs, physical exercise (either strengthening and aerobic resistance training) is considered in CPGs as the management strategy with the highest degree of recommendation, since it improves pain and physical function [22, 23] Although its ideal posology remains unclear, two or three sessions of 30–45 min of physical activity with mild-to-moderate intensity could be an effective strategy [24]. Other strongly recommended physiotherapy approaches are thermal therapy, acupuncture, or hydrotherapy, since their improvement in pain, anxiety, depression, fatigue, sleep, physical function, or quality of life have been explored and confirmed [11, 25–27]. Even though physiotherapy plays a fundamental key role in the management of FM, several treatments do not incorporate physiotherapy, and many patients receiving physiotherapy as a part of the therapeutic strategy disclose having had bad experiences [28]. Numerous barriers have been identified when implementing research into clinical practice: time constraints, low levels of self-efficacy towards evidence-based practice activities, or negative perceptions about research, amongst others [29–31]. This failure to follow evidence-based recommendations could lead to an “evidence-to-practice gap”.

The different approaches of health professionals to FM have been explored. In the UK, the PACFiND study [32] found that, after patient’s education, pharmacological treatment is the most common therapeutical approach used with FM by healthcare professionals from the National Health Services (NHS), while solely 33,33% of them provide non-pharmacological interventions such as structured exercise, psychological therapies, and multicomponent programs. In China, eighty percent of the respondents to the study of Mu et al. [33] declared having difficulties in treating FM patients. Concerning the specific approach focused on physiotherapists, Alodiabi et al. [34] have recently studied the level of knowledge on FM specifically among physiotherapists, evidencing little knowledge and lack of confidence in assessing and managing FM cases. The operational assessment of the professional adherence to CPGs is not standardized; however, recent studies have based its evaluation on a three-section structured survey, encompassing demographic data, coverage of adherence based on clinical cases, and level of agreement with CPG-based statements [35, 36]. To date, no research has delved into the level of knowledge and the degree of adherence to CPGs for FM that physiotherapists have in Spain. Therefore, the main objective of the current study was to evaluate the level of knowledge and adherence to CPGs on FM of physiotherapists in Spain.

## Methods

### Design

A cross-sectional study using an ad-hoc online survey was implemented, with the aim of exploring the level of knowledge and compliance/adherence to CPGs of Spanish physiotherapists with respect to FM [11, 12]. The questionnaire was developed in Spanish (see Annex 1; also provided in English as Supplementary Material—see Annex 2-) according to the recommendations of the International Handbook of Survey Methodology [37]. First, the latest GPCs on the therapeutic management of the condition were identified and explored in depth [11, 12]. Subsequently, the general structure of a three-section survey aiming to collect data on sociodemographic data but, more specifically, to capture the level of adherence and to cover the agreement with the different statements observed by the CPGs was adopted, based on the methodology recently implemented by Battista et al. [35] and Caffini et al. [36] on different conditions, namely osteoarthritis and ankle sprains, respectively. The general recommendations of the STROBE guidelines for observational studies and the CROSS Checklist for Reporting of Survey Studies were followed [38, 39]. This study was carried out following the principles of the Declaration of Helsinki and obtained approval from the Ethics Committee of the University of Murcia under the code 3854/2023.

### Participants

To be included in the study, participants had to provide their consent to participate in the study. Inclusion criteria included current professional activity as a physiotherapist in Spain and having treated at least one subject affected by FM in the 2-year timeframe prior to the study onset. Those professionals having provided at least a negative answer to one of these two questions could not continue the survey and received a thank you message.

Participants were recruited by different sources: contacting them on a one-by-one basis through their professional email addresses (throughout the contact details of the different clinical settings registered on the official website of the professional associations of Physiotherapy of the different Spanish autonomous communities). Also, the hyperlink to the questionnaire was provided through the intermediation of the professional associations to the emails of the registered professionals of 7 of the 17 autonomous communities in Spain. Finally, the authors also encouraged its dissemination on different socio-professional networks.

### Procedure

Data for this study were collected through an electronic ad-hoc survey created with Google Forms, a secure web application for building and managing online surveys and databases in compliance with General European Data Protection Law [40]. Data were collected between September 2023 and January 2024. Prior to answering the survey, a cover letter was included with specific information on the purpose of the study, and participants were provided with the corresponding informed consent.

The questionnaire was divided into three sections (see Annex 1) based on the structure and procedure followed in previous research [35, 36]:

1. Section I (questions 1–12) was subdivided into subsection A, which included information about the study and the informed consent, and subsection B, which requested sociodemographic data.
2. Section II of the survey (questions 13–15) requested professionals on how they would handle a clinical case using clinical vignettes. The clinical case presented a patient diagnosed with FM and provided data on her socio-demographic characteristics and symptoms. The respondent was asked to indicate, out from the options on a list, what aspects he/she would include in the assessment, treatment, and decision of the length of the therapeutic approach. Clinical vignettes are valid and acceptable tools for measuring clinical decision making and adherence to evidence-based practice [41].
3. In Section III (questions 16–39) participants were requested to choose their level of agreement with a series of 24 statements using a 5-point Likert scale, in which “1” meant “completely disagree” and “5” stood for “completely agree”. Participants were considered to agree with the statements whenever the score was 4–5 and, conversely, to disagree with the statements if the score ranged in a 1-to-3 range. Furthermore, to limit acquiescence bias, that is, the tendency to agree with all research statements in the survey [42], seven inverted statements out of a total of 24 were included in section III of the questionnaire, so that disagreement with those statements (scores 1–2) indicated in fact agreement with the CPGs. The questions in section II and III were asked based on the EULAR and SIR CPGs on the diagnosis and management of fibromyalgia [11, 12]. Table 1 displays the different statements and recommendations based on both CPGs.

**Table 1:** Summary of recommendations according to SIR and EULAR

	Level of evidence	
	SIR	EULAR
Assessment and evaluation		
For recommendations		
Assessment of pain, function, comorbidities and psychosocial context	IV	IV
Abidance of laboratory tests and radiographic analysis	V	Undisclosed
Weak against recommendations	Tender points examination	
Strong against recommendations		
Neutral statements (not disclosed): neurological examination, gait and posture assessment		
Treatment and management		
For recommendations		
Global pharmacological treatment	I	**
Physical therapies		
Aerobic resistance training	I	****
Strengthening exercise	I	****
Water activity/water jogging	I	**
Thermal therapy	I	**
Psychological Therapies		
Behavioral-cognitive therapy and occupational therapy	I	**
Therapeutic writing	III	Undisclosed
Non-conventional therapies		
Meditative movement therapies (qigong, yoga, tai chi)	I	**
Mindfulness	I	**
Acupuncture	I	**
Hydrotherapy	I	**
Return to work	V	Undisclosed
Self-management and promotion of self-efficacy	I	Not specifically defined
Patient education	I	**
Weak against recommendations		Biofeedback, massage
Strong against recommendations		Homeopathy, chiropractic
Controversial statements: hypnosis, guided imagination	III	Hypnosis: weak against; guided imagination: strong against
Neutral statements (not disclosed): stretching, postural reeducation, magneto therapy, ultrasounds, electrotherapy, trigger points		

SIR follows the Oxford levels of evidence: I. From meta-analysis of randomised controlled trials or from at least one randomised controlled trial; II. From at least one controlled study without randomisation or from at least one cohort study; III. From at least one case–control study; IV. From case-series or poor-quality cohort and case–control studies; V. From expert committee reports or opinions and/or clinical experience of respected authorities. EULAR follows either the aforementioned Oxford system, or the following one

*It has limited evidence

**It has some evidence

***It has quite a lot of evidence

****It is supported by a lot of evidence

The survey was initially tested by the six authors of the current study and, subsequently, by ten professional physiotherapists specialized in musculoskeletal rehabilitation, so that they could review and make a qualitative assessment of each of the items, the response options, and the structure of the entire content of the questionnaire. After reviewing and assessing the clarity, comprehensibility, and appropriateness for the target-group of the questionnaire, the aforementioned professionals confirmed both face validity (whether a test appears to measure what it is supposed to measure) and content validity (the extent to which and instrument covers all relevant parts of the construct it aims to measure). The professionals took a mean length of 4 min 53 s to implement the questionnaire.

### Variables

The main objective of the current research was to describe the knowledge and adherence to current CPGs and recommendations for Spanish physiotherapists in patients with FM. The responses provided by participants were compared to the EULAR and SIR recommendations [11, 12], to establish the level and extent of agreement. In section II, scores were compared to those from the EULAR and SIR CPGs [11, 12], and subjects were classified as 'adherent' (selection of all the actions recommended by the CPGs and having selected none of those not recommended, i.e., participants were considered as “adherents” whenever all the options marked were indeed recommended -which entails that they could not have marked all the recommended actions, but solely a percentage of the recommended items-), ‘partially adherent’ (whenever participants marked recommended options alongside “neutral statements”) and ‘non-adherent’ (whenever at least one of the “weak against recommendations” or “strong against recommendations” were selected, or if they decided either not to treat the patient or to treat him/her for fewer than five sessions) [35]. Concerning the “controversial statements” found, these were not taken into account, given the divergence of opinion between both CPGs used as the gold standard in the current research.

### Statistical analyses

A descriptive analysis of the characteristics of the sample (section I) was implemented. Frequencies and percentages were displayed for the qualitative variables and mean and standard deviation values were calculated for the quantitative variables.

In section II, participants were classified as ‘adherent’, ‘partially adherent’ and ‘non-adherent’, and the frequencies and descriptive statistics of each subcategory were compared through Chi-square tables and ANOVA tests, respectively.

Concerning section III, participants who totally or partially agreed with a statement (score 4–5) or partially or completely disagreed with the opposite (score 1–2) were considered to globally agree with the recommendations of the CPGs. The overall consensus with each statement was investigated. In the absence of a standard threshold, a ≥ 70% agreement with a statement was adopted as consensus [43].

All analyses were performed using IBM SPSS Statistics for Windows, Version 28.0 (Armonk, NY, USA: IBM Corp; 2016), with a p-level of significance set at p < 0.05.

Sample size was also calculated as of the formula per finite populations [44]. Calculation was based on the current population of physiotherapists in Spain, corresponding to a total number of 62,691 professionals overall [45]. Considering a 95% Confidence Interval, and a 7% margin of error [46], the minimal sample size needed consisted of 196 subjects.

## Results

**Fig. 1:**
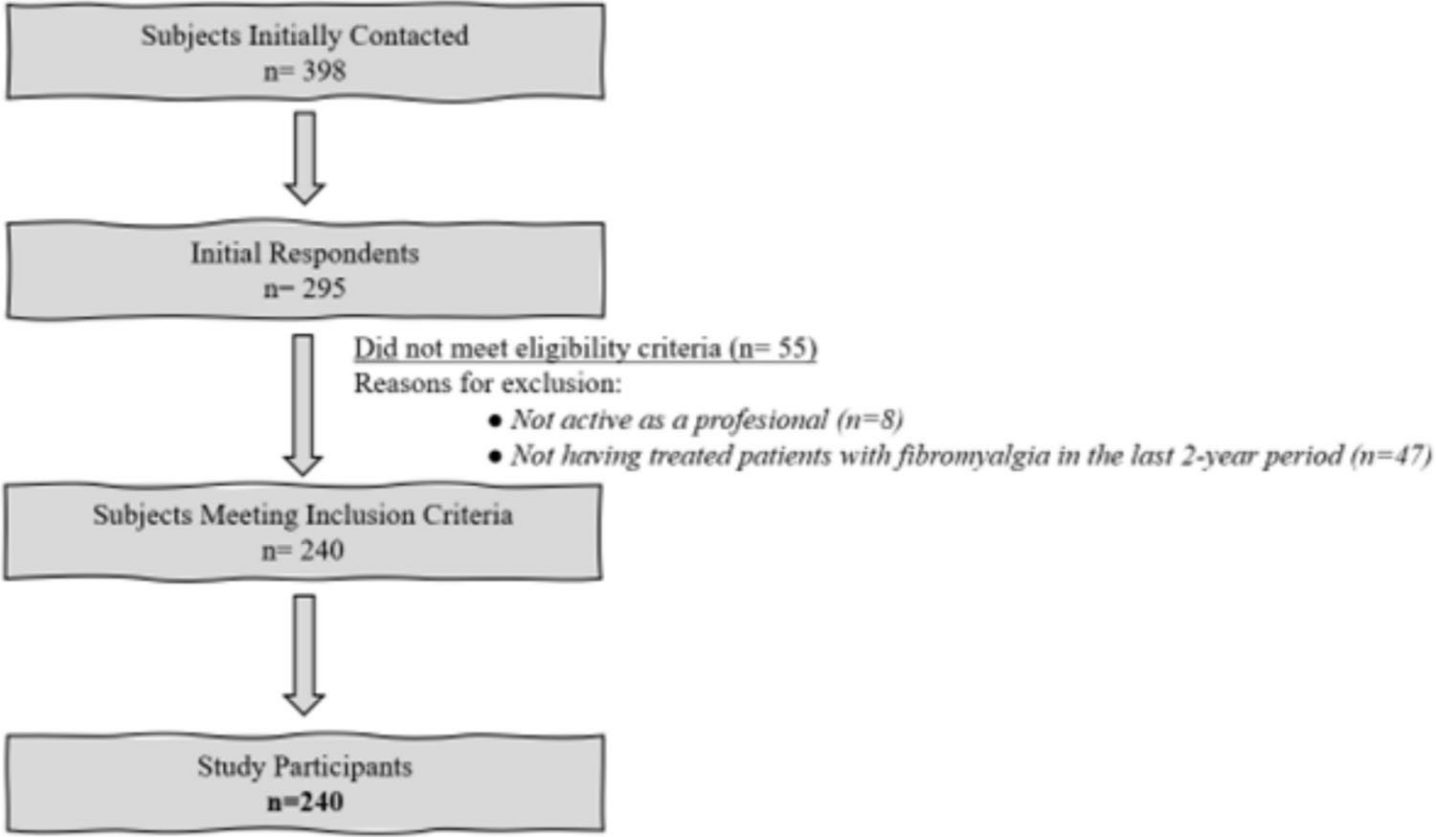
Study flowchart

A total of 295 physiotherapists initially responded, among which 240 (81.35%) met inclusion criteria and were subsequently surveyed of the three sections in our study (see Fig. 1). The total sample surveyed (240 subjects; mean age ± standard deviation = 37.21 ± 12.98) was gender-balanced (106 men, 44.16%; 134 women, 55.83%). Exactly 50% (n = 120) of the sample had had previous experiences with reading and/or applying at least one CPG in the field of FM. Table 2 displays the main characteristics of the participants.

**Table 2:** Participants’ characteristics

Participants’ characteristics (n = 240)	Mean ± standard deviation; frequency (percentage)
Sociodemographic variables	
Age (years)	37.21 ± 12.98
Gender (female)	134 (55.83)
Academic-professional variables	
Professional experience (years)	15.4 ± 7.06
Academic level of studies	
Undergraduate degree	145 (60.41)
Master’s degree	86 (35.83)
PhD	9 (3.75)
Previous training in fibromyalgia (yes)	76 (31.66)
Having previously read clinical practice guideline in fibromyalgia (yes)	120 (50.00)

In Section II of the survey, the ‘adherent’ group (i.e. professionals providing the FM-affected subjects with all treatments included amongst the CPGs recommended treatments) corresponded to 68 subjects of the sample (28.33%). A total of 52 subjects (21.67%) were ‘partially adherent’ (since at least one of the “neutral statements” was included within the assessment -neurological, gait and posture- or management therapeutic strategy -stretching, postural reeducation, magnetotherapy, ultrasounds, electrotherapy, trigger points-), and the extant figures, i.e., a total of 120 subjects (50.00%), corresponded to the ‘non adherent’ group. Amongst them, 13 subjects proposed a therapeutic strategy below 5 sessions, whilst 116 subjects chose massage and 8 physiotherapists specifically indicated chiropractic and/or homeopathic approaches as part of the therapy (the three aforementioned approaches are specifically indicated as “non-recommended” in the current guidelines); with the aim of better understanding the physiotherapists’ beliefs on FM, some questions on the assessment of the condition was also included, and the responses showed a high number of professionals including, for instance, the evaluation of tender points as a useful assessment-based strategy (n = 118; 49.16%), whilst its examination has little clinical relevance and does not confirm a diagnosis of fibromyalgia (level 5 recommendation) [11]. Since some responses overlapped (i.e.: some professionals, for instance, supported and endorsed the use of chiropractic approaches and massage concurrently), the final number of respondents included in the ‘non adherent’ group accounted for 120 subjects overall.

**Fig. 2:**
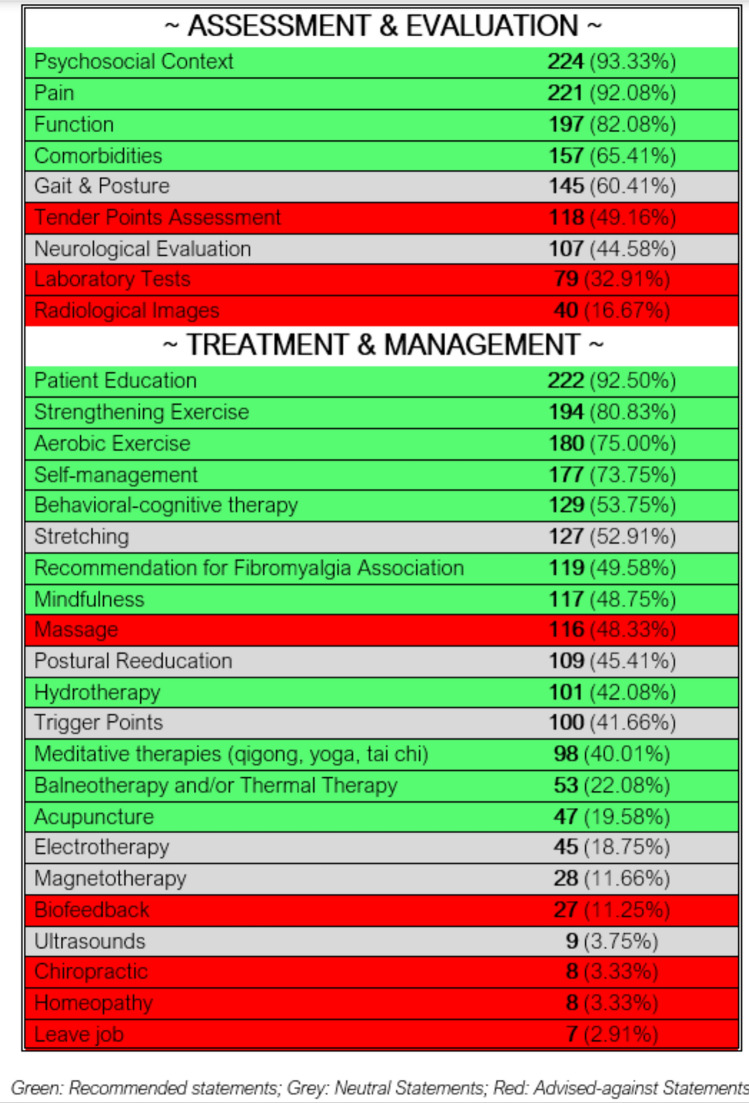
Response rate for each recommendation statement (n = 240)

Patient education (n = 222), strength exercises (n = 194), and aerobic exercise (n = 180) were supported and endorsed by a vast majority of professionals. Therapies as stretching (an actual “neutral statement”) received 127 responses, whilst biofeedback, for instance, expounded a lower number of responses (n = 27). Other therapeutic approaches diverting from the physiotherapy-based daily clinical activity experienced a heterogeneous response rate: cognitive-behavioral therapy accounted for 129 responses. Figure 2 displays the response rate for each one of the different statements explored.

None of the sociodemographic variables differed across the three groups (adherent vs partially adherent vs non-adherent). As for the academic and professional characteristics, the years of professional experience did not account for differences (ANOVA: F = 1.011, p-value = 0.471), whereas both the academic level of studies (Chi-square = 48.601, p-value = 0.001) and having had previous training in FM (Chi-square = 151.011, p-value = 0.001) displayed statistically significant differences across groups.

**Fig. 3:**
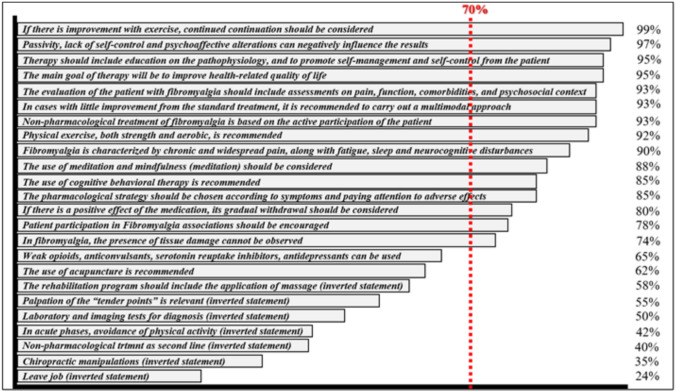
Overall consensus per statement

Focusing on section III, overall consensus (i.e., equal or above 70%) was reached for 15 out of 24 statements. Amongst the nine statements below the threshold of 70%, a total of seven corresponded to inverted statements. Thus, solely two “true” statements did not reach the aforementioned threshold: “Weak opioids (tramadol), anticonvulsants/antiepileptics (pregabalin), serotonin and norepinephrine reuptake inhibitors (fluoxetine, paroxetine and duloxetine), as well as tricyclic antidepressants (amitriptyline) and cyclobezaprine and cannaboids can be used to modulate pain” reached 65% consensus, whereas “The use of acupuncture is recommended” covered a consensus-based percentage of 62%, as shown in Fig. 3.

## Discussion and conclusion

### Discussion

To the best of our knowledge, this is the first study to explore the level of knowledge and adherence to CPGs in the field of FM by physiotherapists in Spain.

In our study, exactly 50% (n = 120) of the sample had had previous experiences with reading and/or applying at least one CPG in the field of FM. This finding is in line with previous research having stated that roughly one physiotherapist out of two follows evidence-based recommendations in clinical practice [47]. Furthermore, among those professionals who adhere to CPGs, the implementation of those strategies is not always adequately channeled, since recommendations from CPGs do not have an automatic translation to clinical practice, as expounded by Kristensen et al. [48], facts that promote the so called “evidence-to-practice gap”.

The level of knowledge and adherence seems to be conditioned by both the academic level achieved and the existence of a previous background and training on FM. Counterintuitively, the years of professional experience do not display a significant impact in this respect. However, although practical experience provides an outstanding substratum for theoretical knowledge [49], the conceptualization of FM has thoroughly and consistently evolved, in the last decades, from a substrate of difficult-to-explain pain mixed with psychosocial symptoms alongside the emergence of multiple criteria to define the disorder, to the current paradigm of the condition [50]. This double context could explain the fact that some professionals with a relevant length of professional experience could subsequently not be updated despite their wide experience.

Concerning the diagnosis of the condition, roughly half of the respondents (n = 118; 49.16%) selected pain on palpation of the tender points as part of the examination, in line with the findings displayed by other authors [32, 33, 51], even though its inclusion as part of the diagnostic criteria has been advised against in the last decades, because of its lack of validity and/or clinical relevance [8, 11, 52]. Even though the specific diagnosis is not a professional competence of physiotherapists, the clear identification of the diagnostic criteria for FM becomes of paramount importance, since they are often first-line healthcare providers, i.e. patients commonly consult a physiotherapist in a “first intention” fashion, which results in a safe, less expensive, and reliable management model of care [53].

Focusing on the therapeutic approach, patient education (n = 222; 92.50%), strength training (n = 194; 80.83%), aerobic exercise (n = 180; n = 75%), and self-management strategies (n = 177; 73.75%) were identified by the vast majority of respondents. These findings are aligned with the statements supported and endorsed by the most recent CPGs [11, 12], and run along similar lines to the findings expounded in previous similar research in the field [34]. On another note, therapies labelled as “neutral” received a heterogeneous level of support, with figures on a 3.75–52.91% range, corresponding to ultrasound and stretching, respectively. An interesting aspect in this framework is the similarity on the percentage of respondents who selected palpation of tender points as an exploration means (n = 118; 49.16%) and the treatment of trigger points as a therapeutic strategy (n = 100, 41.66%). This aspect may reinforce the idea that both entities are frequently mistaken, as stated elsewhere [54, 55]. Approximately 50% of the participants considered massage as a useful therapeutic approach in the management of patients with FM: this high response rate may be influenced by the relevance of massage-therapy across university curricula in Spain. Despite not presenting adverse effects, its application is not recommended, given that no evidence on its potential impact on pain has been found [12]. Finally, techniques with high levels of evidence and recommendation, such as acupuncture or balneotherapy, received low support levels (22.08% and 19.58%, respectively). These results are consistent with those from Alodiabi et al. [34], in which less than 30% of the participants endorsed and supported the use of acupuncture. Furthermore, the management of FM involves other healthcare providers [56] and, consequently, physiotherapists should be aware of potential concomitant aspects (i.e., worsening of symptoms, or mental health issues) to eventually refer the patient to a rheumatologist or psychiatrist for an integral approach.

As far as the consensus of the different statements is concerned, the application of acupuncture and the use of pharmacological treatment, for instance, did not reach the threshold of consensus. The lack of consensus on acupuncture seems congruent and foreseeable in view of the low level of support received by the therapy in the previous section. A feasible explanation lies on the fact that acupuncture is usually excluded from the study plans of the university curricula of Physiotherapy degrees in Spain, and its training is usually provided by means of postgraduate and professional specific courses after graduation [57]. On another note, the lack of consensus concerning the use of pharmacological treatment may respond to a commonly-shared aspect from different Physiotherapy degrees across most universities in Spain: no specific training on pharmacology is approached in the degree, since physiotherapists are solely allowed to indicate, use, and authorize, autonomously, medications not subject to medical prescription, which widely hinders the competence of dispensing and, subsequently, the specific need for a thorough training in this discipline, in contrast to the professional competences of physiotherapists in other health-care systems [58]. However, and despite the fact that pharmacological treatment is not considered the first line of action in the management of FM [11, 12], a deeper understanding of the pharmacological interactions and potential adverse events from physiotherapists would be not only relevant but advisable for a better global management of the condition.

The results stemming from our study should be interpreted in the light of its methodological limitations. First, one of the potential exclusion criteria corresponded to subjects not having treated at least one patient with FM in the last 2 years. This criterion aims to select professionals with a certain recent contact with the condition. However, the fact that professionals with an adequate theoretical and/or academic training on the condition could have been potentially excluded because of their lack of therapeutical experience remains possible. Secondly, the sample size selected may be observed as relatively low, but both the minimum sample size calculated and the representation of 16 out of 19 regions -17 regions plus two autonomous cities- of Spain in the sample support and endorse the inferential potential of the results stemming from the current study. Third, the criterion for classification as “non-adherent”, although congruent with previous research, could be observed as potentially “severe”, since some of the study participants solely selected one non-recommended therapy among a wide range of recommended therapies, fact that made them ‘tilt’ into the “non-adherent” group. Finally, the potential professional-based social desirability response bias was not controlled in the current study. Since this bias is common and inherent to questionnaires and survey-based studies, future research could delve into the methods suggested to avoid or minimize the potential impact of its effects [59].

The main impact stemming from the results of the current study is based on the possibility to objectify the “picture” of the current therapeutic approach to fibromyalgia by physiotherapists in Spain, in a first step to improve the treatment strategies and to enhance the bonds between scientific evidence and clinical practice. Future research should focus on factors facilitating and hindering the implementation of the existing scientific evidence in daily clinical practice in the field of FM. This fact could enhance and improve the general picture of the physical therapy approach of the condition, leading to the implementation of tailored interventions and resulting in better care for patients with fibromyalgia. University-based study plans could also be updated and enhanced in terms of evidence-based practice, to empower new graduates with better knowledge on the management of FM, in accordance with the most current scientific evidence.

## Conclusion

Our findings highlight the presence of an acceptable level of knowledge and adherence on clinical practice guidelines in the field of fibromyalgia among physiotherapists in Spain.

## Practice implications

Our results also reveal the existence of an evidence-to-practice gap in the field, with potential room for improvement. Further efforts on promoting and reinforcing the importance of evidence-based therapies are needed, from university teaching plans to clinical updates for daily practice.

### Supplementary Information

Below is the link to the electronic supplementary material.

Supplementary file1 (DOCX 27 KB)

## Data Availability

Datasets used in the current research are available under the following DOI: 10.7910/DVN/Z2KCKT.

## Declarations

### Identification statement

We confirm all patient/personal identifiers have been removed or disguised so the patient/person(s) described are not identifiable and cannot be identified through the details of the story.

## References

[1] Spaeth M (2009). Epidemiology, costs, and the economic burden of fibromyalgia. Arthritis Res Ther.

[2] Sarzi-Puttini P, Giorgi V, Marotto D (2020). Fibromyalgia: an update on clinical characteristics, aetiopathogenesis and treatment. Nat Rev Rheumatol.

[3] Font GT, Bordoy FC, Juan MA, Seoane-Mato D, Álvarez RF, Delgado SM, Martínez DC, Sánchez-Fernández SA, Marena RVL, García MPV, Olivé A, Rubio MP, Larrosa M, Navarro RN, Sánchez PC, Díaz-González F, Bustabad-Reyes S (2020). Prevalence of fibromyalgia and associated factors in Spain. Clin Exp Rheumatol.

[4] Fitzcharles MA, Cohen SP, Clauw DJ, Littlejohn G, Usui C, Häuser W (2021). Nociplastic pain: towards an understanding of prevalent pain conditions. Lancet.

[5] Harte SE, Harris RE, Clauw DJ (2018). The neurobiology of central sensitization. J Appl Behav Res.

[6] Häuser W, Ablin J, Fitzcharles MA (2015). Fibromyalgia. Nat Rev Dis Primers.

[7] Álvarez-Gallardo IC, Estévez-López F, Torres-Aguilar XC, Segura-Jiménez V, Borges-Cosic M, Soriano-Maldonado A, Camiletti-Moirón D, García-Rodríguez IC, Munguía-Izquierdo D, Sierras-Robles Á, Delgado-Fernández M, Girela-Rejón MJ (2019). Physical activity, sedentary behaviour, physical fitness, and cognitive performance in women with fibromyalgia who engage in reproductive and productive work: the al-Ándalus project. Clin Rheumatol.

[8] Wolfe F, Clauw DJ, Fitzcharles MA, Goldenberg DL, Häuser W, Katz RL, Mease PJ, Russell AS, Russell IJ, Walitt B (2016). Revisions to the 2010/2011 fibromyalgia diagnostic criteria. Semin Arthritis Rheum.

[9] Prikhodkina M, Melnikov S (2024). Factors that influence medication adherence in women with fibromyalgia: a path analysis. J Clin Nurs.

[10] Goldenberg DL, Burckhardt C, Crofford L (2004). Management of fibromyalgia syndrome. JAMA.

[11] Ariani A, Bazzichi L, Sarzi-Puttini P, Salaffi F, Manara M, Prevete I, Bortoluzzi A, Carrara G, Scirè C, Ughi N, Parisi S (2021). The Italian Society for Rheumatology clinical practice guidelines for the diagnosis and management of fibromyalgia. Best practices based on current scientific evidence. Reumatismo.

[12] Macfarlane GJ, Kronisch C, Dean LE, Atzeni F, Häuser W, Fluß E, Choy E, Kosek E, Amris K, Branco J, Dincer F, Leino-Arjas P, Longley K, McCarthy GM, Makri S, Perrot S, Sarzi-Puttini P, Taylor A, Jones GT (2017). EULAR revised recommendations for the management of fibromyalgia. Ann Rheum Dis.

[13] Diviney M, Dowling M (2015). Lived experiences of FM. Primary Health Care.

[14] Byrne A, Jones K, Backhouse M, Rose F, Moatt E, van der Feltz-Cornelis C (2023). Patient and primary care practitioners' perspectives on consultations for fibromyalgia: a qualitative evidence synthesis. Prim Health Care Res Dev.

[15] Amutio A, Franco C, Sánchez-Sánchez LC, Pérez-Fuentes MDC, Gázquez-Linares JJ, Van Gordon W, Molero-Jurado MDM (2018). Effects of mindfulness training on sleep problems in patients with fibromyalgia. Front Psychol.

[16] Pleman B, Park M, Han X, Price LL, Bannuru RR, Harvey WF, Driban JB, Wang C (2019). Mindfulness is associated with psychological health and moderates the impact of fibromyalgia. Clin Rheumatol.

[17] Williams AC, Eccleston C, Morley S (2012). Psychological therapies for the management of chronic pain (excluding headache) in adults. Cochrane Database Syst Rev.

[18] Kachaner A, Harim M, Combier A, Trouvin AP, Avouac J, Ranque B, Piot MA (2023). Management perspectives from patients with fibromyalgia experiences with the healthcare pathway: a qualitative study. Front Med (Lausanne).

[19] Migliorini F, Maffulli N, Eschweiler J, Knobe M, Tingart M, Colarossi G (2022). Pharmacological management of fibromyalgia: a Bayesian network meta-analysis. Expert Rev Clin Pharmacol.

[20] Farag HM, Yunusa I, Goswami H, Sultan I, Doucette JA, Eguale T (2022). Comparison of amitriptyline and US Food and Drug Administration-approved treatments for fibromyalgia: a systematic review and network meta-analysis. JAMA Netw Open.

[21] Lederman S, Arnold LM, Vaughn B, Kelley M, Sullivan GM (2023). Efficacy and safety of sublingual cyclobenzaprine for the treatment of fibromyalgia: results from a randomized, double-blind. Placebo-Controlled Trial Arthritis Care Res (Hoboken).

[22] Albuquerque ML, Monteiro D, Marinho DA (2022). Effects of different protocols of physical exercise on fibromyalgia syndrome treatment: systematic review and meta-analysis of randomized controlled trials. Rheumatol Int.

[23] Chen J, Han B, Wu C (2022). On the superiority of a combination of aerobic and resistance exercise for fibromyalgia syndrome: a network meta-analysis. Front Psychol.

[24] Sosa-Reina MD, Nunez-Nagy S, Gallego-Izquierdo T, Pecos-Martín D, Monserrat J, Álvarez-Mon M (2017). Effectiveness of therapeutic exercise in fibromyalgia syndrome: a systematic review and meta-analysis of randomized clinical trials. Biomed Res Int.

[25] Honda Y, Sakamoto J, Hamaue Y, Kataoka H, Kondo Y, Sasabe R, Goto K, Fukushima T, Oga S, Sasaki R, Tanaka N, Nakano J, Okita M (2018). Effects of physical-agent pain relief modalities for fibromyalgia patients: a systematic review and meta-analysis of randomized controlled trials. Pain Res Manag.

[26] Valera-Calero JA, Fernández-de-Las-Peñas C, Navarro-Santana MJ, Plaza-Manzano G (2022). Efficacy of dry needling and acupuncture in patients with fibromyalgia: a systematic review and meta-analysis. Int J Environ Res Public Health.

[27] Ma J, Zhang T, Li X, Chen X, Zhao Q (2024). Effects of aquatic physical therapy on clinical symptoms, physical function, and quality of life in patients with fibromyalgia: a systematic review and meta-analysis. Physiother Theory Pract.

[28] Lempp HK, Hatch SL, Carville SF (2009). Patients' experiences of living with and receiving treatment for fibromyalgia syndrome: a qualitative study. BMC Musculoskelet Disord.

[29] Fruth SJ, Van Veld RD, Despos CA, Martin RD, Hecker A, Sincroft EE (2010). The influence of a topic-specific, research-based presentation on physical therapists' beliefs and practices regarding evidence-based practice. Physiother Theory Pract.

[30] Gleadhill C, Bolsewicz K, Davidson SRE (2022). Physiotherapists’ opinions, barriers, and enablers to providing evidence-based care: a mixed-methods study. BMC Health Serv Res.

[31] Stander J, Grimmer K, Brink Y (2021). Time as a barrier to evidence uptake-a qualitative exploration of the concept of time for clinical practice guideline uptake by physiotherapists. J Eval Clin Pract.

[32] Wilson N, Beasley MJ, Pope C (2022). UK healthcare services for people with fibromyalgia: results from two web-based national surveys (the PACFiND study). BMC Health Serv Res.

[33] Mu R, Li C, Zhu JX, Zhang XY, Duan TJ, Feng M, Wang GC, Zhang FC, Li ZG (2013). National survey of knowledge, attitude and practice of fibromyalgia among rheumatologists in China. Int J Rheum Dis.

[34] Alodiabi F, Alhowimel A, Alotaibi M, Alamam D, Fritz JM (2020). Knowledge, awareness, and perceptions of the diagnosis and management of fibromyalgia among physical therapists in Saudi Arabia: a cross-sectional survey. Open Access Rheumatol.

[35] Battista S, Salvioli S, Millotti S, Testa M, Dell'Isola A (2021). Italian physiotherapists' knowledge of and adherence to osteoarthritis clinical practice guidelines: a cross-sectional study. BMC Musculoskelet Disord.

[36] Caffini G, Battista S, Raschi A, Testa M (2022). Physiotherapists' knowledge of and adherence to evidence-based practice guidelines and recommendations for ankle sprains management: a cross-sectional study. BMC Musculoskelet Disord.

[37] de Leeuw D, Hox JDD (2008). International Handbook of Survey Methodology (European Association of Methodology Series).

[38] von Elm E, Altman DG, Egger M, Pocock SJ, Gøtzsche PC, Vandenbroucke JP (2008). The Strengthening the Reporting of Observational Studies in Epidemiology (STROBE) statement: guidelines for reporting observational studies. J Clin Epidemiol.

[39] Sharma A, Minh Duc NT, Luu Lam Thang T, Nam NH, Ng SJ, Abbas KS, Huy NT, Marušić A, Paul CL, Kwok J, Karbwang J, de Waure C, Drummond FJ, Kizawa Y, Taal E, Vermeulen J, Lee GHM, Gyedu A, To KG, Verra ML, Jacqz-Aigrain ÉM, Leclercq WKG, Salminen ST, Sherbourne CD, Mintzes B, Lozano S, Tran US, Matsui M, Karamouzian M (2021). A Consensus-Based Checklist for Reporting of Survey Studies (CROSS). J Gen Intern Med.

[40] Blackmer WS (2018) EU general data protection regulation. Am Fuel Petrochemical Manuf AFPM. In: Labor Relations/Human Resour Conference, pp 45–62

[41] Ladeira CE, Cheng MS, Da Silva RA (2017). Clinical specialization and adherence to evidence-based practice guidelines for low back pain management: a survey of US physical therapists. J Orthop Sports Phys Ther.

[42] Suárez-Alvarez J, Pedrosa I, Lozano LM, García-Cueto E, Cuesta M, Muñiz J (2018). Using reversed items in Likert scales: a questionable practice. Psicothema.

[43] Teo PL, Hinman RS, Egerton T, Dziedzic KS, Bennell KL (2019). Identifying and prioritizing clinical guideline recommendations most relevant to physical therapy practice for hip and/or knee osteoarthritis. J Orthop Sport Phys Ther.

[44] Kotrlik J, Higgins C (2001). Organizational research: determining appropriate sample size in survey research appropriate sample size in survey research. Inf Technol Learn Perform J.

[45] Instituto Nacional de Estadística (Spanish National Institute for Statistics): https://www.ine.es/. Accessed 28 Jan 2024

[46] McDonald MP (2005) Validity, data sources. In: Encyclopedia of social measurement. Elsevier, Amsterdam, pp 939–948, ISBN 9780123693983. 10.1016/B0-12-369398-5/00046-3

[47] Zadro J, O’Keeffe M, Maher C (2019). Do physical therapists follow evidence-based guidelines when managing musculoskeletal conditions?. Syst Rev BMJ Open.

[48] Kristensen N, Nymann C, Konradsen H (2015). Implementing research results in clinical practice- the experiences of healthcare professionals. BMC Health Serv Res.

[49] Leask R, Cronje T, Holm DE, Van Ryneveld L (2020). The impact of practical experience on theoretical knowledge at different cognitive levels. J S Afr Vet Assoc.

[50] Wolfe F, Rasker JJ (2021). The evolution of fibromyalgia, its concepts, and criteria. Cureus.

[51] Agarwal A, Oparin Y, Glick L, Fitzcharles MA, Adachi JD, Cooper MD, Gallo L, Wong L, Busse JW (2018). Attitudes toward and management of fibromyalgia: A National Survey of Canadian Rheumatologists and Critical Appraisal of Guidelines. J Clin Rheumatol.

[52] Wolfe F, Clauw DJ, Fitzcharles MA, Goldenberg DL, Häuser W, Katz RS, Mease P, Russell AS, Russell IJ, Winfield JB (2011). Fibromyalgia criteria and severity scales for clinical and epidemiological studies: a modification of the ACR preliminary diagnostic criteria for fibromyalgia. J Rheumatol.

[53] Gallotti M, Campagnola B, Cocchieri A, Mourad F, Heick JD, Maselli F (2023). Effectiveness and consequences of direct access in physiotherapy: a systematic review. J Clin Med.

[54] Bourgaize S, Newton G, Kumbhare D, Srbely J (2018). A comparison of the clinical manifestation and pathophysiology of myofascial pain syndrome and fibromyalgia: implications for differential diagnosis and management. J Can Chiropr Assoc.

[55] Häuser W, Perrot S, Sommer C, Shir Y, Fitzcharles MA (2017). Diagnostic confounders of chronic widespread pain: not always fibromyalgia. Pain Rep.

[56] Rico-Villademoros F, Postigo-Martin P, Garcia-Leiva JM, Ordoñez-Carrasco JL, Calandre EP (2020). Patterns of pharmacologic and non-pharmacologic treatment, treatment satisfaction and perceived tolerability in patients with fibromyalgia: a patients' survey. Clin Exp Rheumatol.

[57] Leirós-Rodríguez R, Arce ME, García-Soidán JL (2015). Current status of postgraduate education in physiotherapy. Educ Med.

[58] Noblet T, Marriott J, Hensman-Crook A, O'Shea S, Friel S, Rushton A (2020). Independent prescribing by advanced physiotherapists for patients with low back pain in primary care: a feasibility trial with an embedded qualitative component. PLoS ONE.

[59] Bernardi R, Nash J (2022). The importance and efficacy of controlling for social desirability response bias. Ethics Behav.

