# Supplementary material for: Physiotherapists’ adherence to Clinical Practice Guidelines in fibromyalgia: a cross-sectional online survey
Source: Rheumatol Int. 2024 Jun 5;44(8):1509–20. doi: 10.1007/s00296-024-05630-4 (PMC11222258; doi:10.1007/s00296-024-05630-4)
Supplement: Supplementary file 1 — Supplementary file1 (DOCX 27 KB) [file 296_2024_5630_MOESM1_ESM.docx]

**SUPPLEMENTARY MATERIAL**

**Annex 1. Original Questionnaire**

*Por favor, lea con detenimiento las siguientes afirmaciones y marque, de forma sincera, su grado de acuerdo con cada una de ellas de 1 (completamente en desacuerdo) a 5 (completamente de acuerdo). Marque solamente una opción por afirmación. Señale la respuesta que mejor refleje su primera reacción, no emplee mucho tiempo con cada pregunta.*

| Afirmación | 1. Completamente en desacuerdo | 2.  Parcialmente en desacuerdo | 3.  Ni acuerdo ni en desacuerdo | 4.  Parcialmente de acuerdo | 5. Completamente de acuerdo |
| --- | --- | --- | --- | --- | --- |
| La fibromialgia es una patología caracterizada por dolor crónico y generalizado, junto con otros síntomas como fatiga, alteraciones del sueño y neurocognitivas. |  |  |  |  |  |
| En la fibromialgia no se puede objetivar la existencia de daño tisular. |  |  |  |  |  |
| La evaluación del paciente con fibromialgia debería incluir: dolor, función, comorbilidades y contexto psicosocial. |  |  |  |  |  |
| De forma general, serán necesarias pruebas de laboratorio y de imagen para poder llegar a un diagnóstico de fibromialgia (afirmación invertida). |  |  |  |  |  |
| La palpación de los “tender points” es relevante en el diagnóstico de fibromialgia así como en la monitorización de la evolución (afirmación invertida). |  |  |  |  |  |
| El principal objetivo de la terapia será mejorar la calidad de vida relacionada con la salud. |  |  |  |  |  |
| La estrategia farmacológica debe escogerse según sintomatología y prestando atención a efectos adversos. |  |  |  |  |  |
| En casos con escasa mejoría al tratamiento estándar, se recomienda llevar a cabo abordaje multimodal (actividad física más, al menos, psicoterapia) compartido con el paciente. |  |  |  |  |  |
| Opioides débiles (tramadol), anticonvulsivos/antiepilépticos (pregabalina), los inhibidores de la recaptación de serotonina y norepinefrina (fluoxetina, paroxetina y duloxetina), además de antidepresivos tricíclicos (amitriptilina) y ciclobezaprina y cannaboides pueden ser utilizados para modular el dolor. |  |  |  |  |  |
| Si hay efecto positivo de la medicación, debería plantearse su retirada gradualmente. |  |  |  |  |  |
| El tratamiento no farmacológico de la fibromialgia constituye la segunda línea de actuación (afirmación invertida). |  |  |  |  |  |
| El tratamiento no farmacológico de la fibromialgia se basa en la participación activa del paciente. |  |  |  |  |  |
| Se debe alentar la participación del paciente en asociaciones de Fibromialgia. |  |  |  |  |  |
| El ejercicio físico, tanto de fuerza como aeróbico, está recomendado. |  |  |  |  |  |
| Si hay mejoría con el ejercicio, debería plantearse la continuación de forma constante. |  |  |  |  |  |
| El programa de rehabilitación debería incluir la aplicación de masaje (afirmación invertida). |  |  |  |  |  |
| El uso de acupuntura está recomendado. |  |  |  |  |  |
| La terapia debe incluir educación sobre fisiopatología de la enfermedad, además de promover el automanejo y el autocontrol por parte del paciente. |  |  |  |  |  |
| En fases agudas debemos recomendar el reposo, evitando la actividad física (afirmación invertida). |  |  |  |  |  |
| Se debe considerar el uso de meditación y mindfulness. |  |  |  |  |  |
| A nivel de psicoterapia, se recomienda el uso de terapia cognitivo conductual. |  |  |  |  |  |
| La intervención terapéutica debe incluir manipulaciones quiroprácticas (afirmación invertida). |  |  |  |  |  |
| Factores como pasividad, falta de autocontrol y alteraciones psicoafectivas pueden influir negativamente en los resultados. |  |  |  |  |  |
| Se recomendará al paciente con fibromialgia que deje su tarea laboral (en caso de encontrarse en activo) (afirmación invertida). |  |  |  |  |  |

**Caso clínico (investigación sobre adherencia)**

*Por favor, lea con detenimiento el siguiente caso clínico e indique a continuación qué técnicas elegiría para abordar al paciente indicado.*

Antonia, mujer de 59 años, diagnosticada de fibromialgia, vive con su marido y sus dos hijos de 27 y 25 años. Presenta ligero sobrepeso (IMC 26.4). Trabaja como comercial desde hace 20 años. Dice que, aunque su trabajo le genera estrés y ansiedad, lo lleva bien. Acude a consulta porque, desde hace 1 año, refiere dolor generalizado. Dice que estos dolores varían en intensidad a lo largo del tiempo. También refiere que se nota más cansada de lo habitual, que el sueño no es reparador, y que tiene molestias gastrointestinales difusas.

A. Indique qué aspectos incluiría en la valoración:

● “Tender points”.

● Dolor.

● Exploración neurológica.

● Función.

● Exploración postura y marcha.

● Comorbilidades.

● Pruebas de laboratorio

● Contexto psicosocial.

● Imágenes radiológicas.

B. Indique qué técnicas de tratamiento incluiría:

● Instrucciones para automanejo.

● Educar al paciente en su patología.

● Ejercicio de fuerza.

● Estiramientos.

● Ejercicio aeróbico.

● Acupuntura.

● Masoterapia.

● Quiropráctica.

● Hidroterapia/hidrocinesiterapia.

● Balneoterapia/terapia termal.

● Ultrasonidos.

● TENS.

● Biofeedback.

● Mindfulness.

● Yoga-Taichí.

● Tratamiento de puntos gatillo (presión inhibitoria, punción seca, etc).

C. Indica cuántas sesiones de tratamiento aplicarías (en total):

● <5.

● 5-10.

● >10.

**Annex 2. English Translated Questionnaire**

*Please read the following statements carefully and honestly mark your level of agreement with each of them from 1 (completely disagree) to 5 (completely agree). Check only one option per statement. Mark the answer that best reflects your first reaction; do not spend too much time on each question.*

| Statement | 1.  Completely disagree | 2.  Partially disagree | 3.  Neither agree nor disagree | 4.  Partially agree | 5.  Completely agree |
| --- | --- | --- | --- | --- | --- |
| Fibromyalgia is a pathology characterized by chronic and widespread pain, along with other symptoms such as fatigue, sleep and neurocognitive disturbances. |  |  |  |  |  |
| In fibromyalgia, the presence of tissue damage cannot be observed. |  |  |  |  |  |
| The evaluation of the patient with fibromyalgia should include assessments on pain, function, comorbidities, and psychosocial context. |  |  |  |  |  |
| In general, laboratory and imaging tests will be necessary to reach a diagnosis of fibromyalgia. (inverted statement). |  |  |  |  |  |
| Palpation of the “tender points” is relevant in the diagnosis of fibromyalgia as well as in monitoring the evolution. (inverted statement). |  |  |  |  |  |
| The main goal of therapy will be to improve health-related quality of life. |  |  |  |  |  |
| The pharmacological strategy should be chosen according to symptoms and paying attention to adverse effects. |  |  |  |  |  |
| In cases with little improvement from the standard treatment, it is recommended to carry out a multimodal approach (physical activity combined with, at least, psychotherapy) taking the patient's preferences into account. |  |  |  |  |  |
| Weak opioids (tramadol), anticonvulsants/antiepileptics (pregabalin), serotonin and norepinephrine reuptake inhibitors (fluoxetine, paroxetine and duloxetine), as well as tricyclic antidepressants (amitriptyline) and cyclobezaprine and cannaboids can be used to modulate pain. |  |  |  |  |  |
| If there is a positive effect of the medication, its gradual withdrawal should be considered. |  |  |  |  |  |
| The non-pharmacological treatment of fibromyalgia constitutes the second line of action. (inverted statement). |  |  |  |  |  |
| Non-pharmacological treatment of fibromyalgia is based on the active participation of the patient. |  |  |  |  |  |
| Patient participation in Fibromyalgia associations should be encouraged. |  |  |  |  |  |
| Physical exercise, both strength and aerobic, is recommended. |  |  |  |  |  |
| If there is improvement with exercise, continued continuation should be considered. |  |  |  |  |  |
| The rehabilitation program should include the application of massage. (inverted statement). |  |  |  |  |  |
| The use of acupuncture is recommended. |  |  |  |  |  |
| Therapy should include education on the pathophysiology of the disease, in addition to promoting self-management and self-control from the patient. |  |  |  |  |  |
| In acute phases we must recommend rest, avoiding physical activity. (inverted statement). |  |  |  |  |  |
| The use of meditation and mindfulness (meditation) should be considered. |  |  |  |  |  |
| The use of cognitive behavioral therapy is recommended. |  |  |  |  |  |
| The therapeutic management should include chiropractic manipulations (inverted statement). |  |  |  |  |  |
| Factors such as passivity, lack of self-control and psychoaffective alterations can negatively influence the results. |  |  |  |  |  |
| The patient with fibromyalgia will be recommended to leave his job (if he/she is active). (inverted statement). |  |  |  |  |  |

**Clinical case (adherence research)**

*Please read the following clinical case carefully and indicate below how you would approach the indicated patient.*

Antonia, a 59-year-old woman, diagnosed with fibromyalgia, lives with her husband and two children, aged 27 and 25. She is slightly overweighted (BMI 26.4). She has been reporting generalized pain for years. She says that this pain varies in intensity over time. She also reports that she feels more tired than usual (she has to take breaks when carrying out her daily activities), that her sleep is not restful, and that she has diffuse gastrointestinal discomfort. She has worked as a salesperson for 20 years. She says that, although her job causes her stress and anxiety, she handles it well.

A. Please indicate what aspects you would include in the assessment. Mark as many as you think as appropriate:

● “Tender points”.

● Pain.

● Neurological examination.

● Function.

● Posture and gait exploration.

● Comorbidities.

● Lab tests.

● Psychosocial context.

● Radiological images.

B. Please indicate what treatment techniques you would include or what recommendations you would give to the patient. Check as many as you think as appropriate.

● Instructions for self-management.

● Educate the patient about her pathology.

● Promote self-efficacy.

● Strength exercise.

● Aerobic exercise.

● Stretching.

● Acupuncture.

● Postural correction.

● Massage therapy.

● Chiropractic

● Hydrotherapy-hydro kinesitherapy.

● Balneotherapy-thermal therapy.

● Magnetotherapy.

● Ultrasound.

● TENS.

● Guided imagery.

● Recommend fibromyalgia association.

● Cognitive behavioral therapy.

● Homeopathy.

● Biofeedback.

● Mindfulness (meditation).

● Yoga-Tai Chi.

● Treatment of trigger points (inhibitory pressure, dry needling, etc.).

● Leave the job.

C. Please indicate how many treatment sessions you would perform (in total).

● <5.

● 5-10.

● >10.
